# Identification of Key Candidate Genes for Beak Length Phenotype by Whole-Genome Resequencing in Geese

**DOI:** 10.3389/fvets.2022.847481

**Published:** 2022-03-15

**Authors:** Jianhua Huang, Cong Wang, Jing Ouyang, Hongbo Tang, Sumei Zheng, Yanpeng Xiong, Yuren Gao, Yongfei Wu, Luping Wang, Xueming Yan, Hao Chen

**Affiliations:** College of Life Science, Jiangxi Science and Technology Normal University, Nanchang, China

**Keywords:** Xingguo gray geese, beak length, whole-genome resequencing, GWAS, *LGI2*

## Abstract

The domestic goose is an important economic animal in agriculture and its beak, a trait with high heritability, plays an important role in promoting food intake and defending against attacks. In this study, we sequenced 772 420-day-old Xingguo gray geese (XGG) using a low-depth (~1 ×) whole-genome resequencing strategy. We detected 12,490,912 single nucleotide polymorphisms (SNPs) using the standard GATK and imputed with STITCH. We then performed a genome-wide association study on the beak length trait in XGG. The results indicated that 57 SNPs reached genome-wide significance levels for the beak length trait and were assigned to seven genes, including *TAPT1, DHX15, CCDC149, LGI2, SEPSECS, ANAPC4*, and *Slc34a2*. The different genotypes of the most significant SNP (top SNP), which was located upstream of *LGI2* and explained 7.24% of the phenotypic variation in beak length, showed significant differences in beak length. Priority-based significance analysis concluded that *CCDC149, LGI2*, and *SEPSECS* genes in the most significant quantitative trait locus interval were the most plausible positional and functional candidate genes for beak length development in the XGG population. These findings not only enhance our understanding of the genetic mechanism of the beak length phenotype in geese, but also lay the foundation for further studies to facilitate the genetic selection of traits in geese.

## Introduction

As a globally important poultry species, geese provide abundant amounts of meat, eggs, and feather products with relatively high economic value for human consumption, especially in China. Currently, China is the leading producer of geese because they have the greatest numbers of geese raised worldwide. They are also the most abundant source of goose breeds in the world (1). The Xingguo gray goose (XGG) is a famous medium-sized gray goose breed in China, which has been included in the conservation list of livestock and poultry genetic resources by China's Ministry of Agriculture. XGG is so named because of its gray feathers and Xingguo County origin. Furthermore, it is known for its high-quality meat and possesses qualities geared toward excellent growth performance, including fast growth, tolerance to roughage, and strong stress resistance.

It is generally believed that facial phenotypes in animals are important because of their close relationship with identification and their morphology is strongly influenced by genetic factors (2). The beak is one of the major structures formed during facial development and it is involved in many significant biological functions, such as predation, vocalization, mating, and grooming. These functions are necessary for adaptation through natural selection and diversifying evolution. Underlying genetic variations might explain the diversity in beak shape, which is a key phenotypic trait. For example, it was found that the *ALX1* and *HMGA2* genes had a major influence on beak shape and size among Darwin's finches (3, 4). In addition, a study on the morphological development of beaks in chickens and ducks suggested that localized cell proliferation mediated by *BMP4* is likely to modulate the size of beaks (5). However, to date, such reports on the beak length of birds, such as domestic geese, have been relatively scarce.

A genome-wide association study (GWAS) is a powerful method for identifying the genetic variations responsible for particular phenotypes. Similarly, the combination of whole-genome resequencing (WGR) technology is expected to provide higher marker density and reveal a wider range of genetic variation than other molecular marker technologies, and enhance the ability to identify the genetic basis of phenotypic traits (6). The increasing availability of WGR provides an effective means for revealing the genetic mechanism of phenotypic traits in detail, as well as a good means for animal protection and breeding. So far, GWAS based on whole-genome resequencing data has been fully applied in many livestock and poultry animals, including pigs (7), cattle (8), chickens (9), and ducks (10).

In general, goose beaks are long, wide, and nearly flat, mainly consisting of upper and lower jaws, with blunt rounded ends, along with numerous horizontal ridges on the edges that are convenient for filtering water and crushing food when eating in water (Supplementary Figure S1). As it is the main organ for ingesting nutrients, studying the genetic mechanism of beak development is of great significance for improving the growth performance and breeding management of geese. In this study, we performed a GWAS on the beak length trait in XGG, based on SNP data obtained by whole-genome resequencing. Through this study, we hope to identify the candidate genes affecting the development of goose beak length, reveal the genetic mechanisms involved, and provide a reference for the study of similar traits in birds.

## Materials and Methods

### Ethics Statement

All procedures used in this study, and those involving animals, complied with the guidelines for the care and utility of experimental animals, which were established by China's Ministry of Agriculture. The ethics committee of Jiangxi Science &Technology Normal University approved this study.

### Experimental Animals

**Figure 1:**
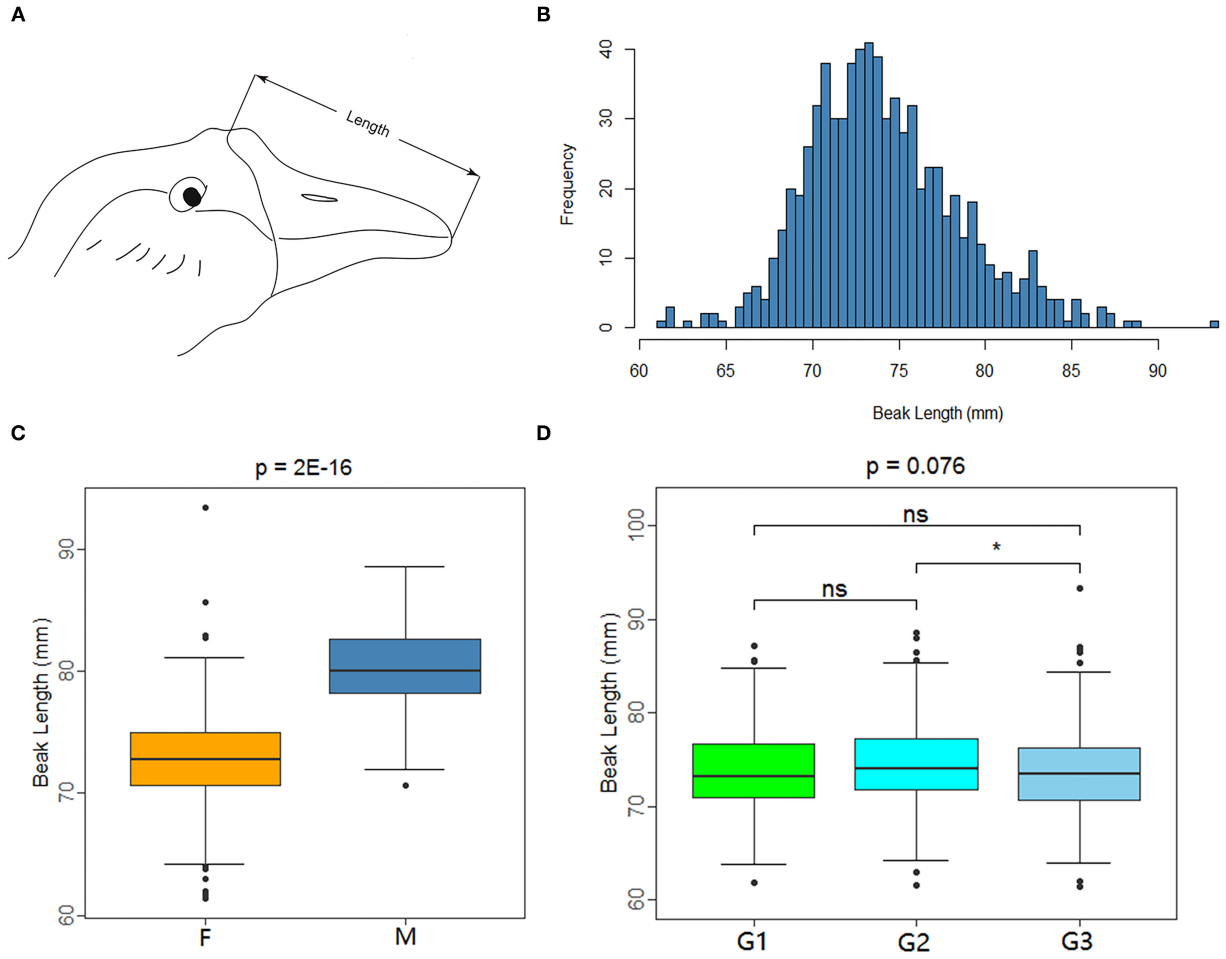
Measuring method and data characteristics of beak length in XGG population. **(A)** Beak length measurement diagram. **(B)** Histogram of the frequency distribution of beak length. **(C,D)** Are box plots of factors associated with beak length measurement. F and M represent female and male **(C)**, and G1, G2, and G3 represent the three measurement groups **(D)**. *Indicates significance (*P* < 0.05), whereas “ns” indicates no significance.

XGG is a nationally protected livestock and poultry breed in China. In the XGG conservation farm, the beaks of the XGG population members show differences in length. To explore the molecular mechanism of beaks, we collected 772 mature XGG samples from animals that were 420-day-old from the Xingguo Gray Goose Farm in Jiangxi Province. Vernier calipers were used to measure the beak lengths of each individual using three different phenotypic measurement groups under the same criteria. The measurement range of beak length was the straight line distance from the feather on the top of the head to the tip of the upper beak (Figure 1A), which was accurate to 0.01 mm. Additionally, vacuum vascular sampling was used to collect 1 mL of blood from the subalar vein of the experimental birds for DNA extraction.

### DNA Extraction and Whole-Genome Resequencing

A modified phenol-chloroform method (11) was used to extract genomic DNA from the fresh blood samples of the experimental birds. The concentration and quality of the DNA samples were assessed using a NanoDrop 2,000 ultra-micro spectrophotometer and 1% agarose gel electrophoresis. Only qualified DNA samples were used to construct paired-end libraries according to standard protocols, with an average insert size of ~350 bp. All libraries were sequenced on the Illumina NovaSeq 6,000 platform to generate raw reads with an average length of ~150 bp. After sequencing, we filtered the raw data to remove adapter sequences and low-quality bases with Trimmomatic (12), resulting in an average depth of ~1 × coverage for clean reads.

### Variant Calling and Quality Control

To obtain a high-quality SNP dataset, 150-bp paired-end clean reads were mapped to the XGG reference genome (https://bigd.big.ac.cn/gwh/, accession number: GWHBAAW00000000) with Burrows–Wheeler alignment MEM (BWA-MEM) (13), using default parameters. Thereafter, the mapping results were converted into BAM format and sorted using SAMtools (14). Duplicate reads were removed using SENTIEON commercial software (15) and calibrated according to the quality score. After mapping, we performed SNP calling for all samples using GATK v4.0.12 (16) and the output was further filtered using PLINK v1.90 (17). The genotype imputation of each individual SNP was completed using STITCH v1.68 (18), and 12,490,912 SNPs were retained with a minimum allele frequency (MAF) of >0.005 and call rate of >0.95. Further quality control methods were applied to the identified SNPs according to the following standards: those with a MAF of less than 0.02 and a missing genotype rate higher than 0.05 were filtered. After a series of quality controls, we obtained a new dataset that consisted of 772 XGG with a total of 11,513,753 high-confidence whole-genome SNPs. These were used, with measured beak length phenotype data, for subsequent genome-wide association analysis.

### Genome-Wide Association Analysis

Here, we used the fastGWA software (19) to implement a GWAS using a mixed linear model as follows:


$$\text{y = }{\text{x}}_{\text{snp}}{\text{β}}_{\text{snp}}\text{ + }{\text{X}}_{\text{c}}{\text{β}}_{\text{c}}\text{+ g + e}$$

where y is an n × 1 vector of phenotypes; x_snp_ is a vector of genotype variables that affect β_snp_; X_c_ is the incidence matrix of fixed effects with their corresponding coefficients β_c_; g is a vector of the total genetic effects with g ~ N (0, $\pi \sigma_{g}^{2}$); π represents the family relatedness matrix (FAM) or the genetic relationship matrix (GRM); e is a vector of residuals with e ~ N (0, ${I\sigma }_{e}^{2}$); and $\pi \sigma_{g}^{2}+{I\sigma }_{e}^{2}$ is the variance-covariance matrix (V) of y.

To enhance the statistical power and minimize false-positive results, we included goose gender and the phenotype measurement groups as fixed effects into the GWAS model. The association significance threshold between SNPs and phenotypes was obtained by Bonferroni adjustment, and the genome-wide significance threshold was set as 0.05/N, which is ~4.34E−09, where N is the number of all SNPs tested. Thereafter, the threshold was extended by 20 times as the chromosome level threshold, namely 1/N, which is ~8.69E−08. To avoid population stratification misleading the validity of the GWAS results, the quantile-quantile plot (Q-Q plot) was used to evaluate the GWAS results and determine whether the calculated *P*-value deviated from the hypothesis test as a whole.

The heritability of the whole genome in this study was calculated based on V_g_/V_p_, where V_g_ and V_p_ represent genetic variance and phenotypic variance, respectively, and V_p_ is the sum of the V_g_ and environmental variance (V_e_). These variances were obtained by constructing a sparse GRM with a cutoff of 0.05 using fastGWA software. Additionally, an ordinary linear model was established to estimate phenotypic variation explained (PVE) by the lead SNP, where PVE is the ratio of the genotype effect variance of the lead SNPs to the sum of the genotype and residual variances in the model.

### Identification of Candidate Genes

The SNPs were annotated using snpEff v4.3t (20) based on the XGG reference genome. Significant SNPs were screened according to the defined significance threshold, and related candidate genes were identified based on the results of variant annotations. The quantitative trait locus (QTL) was defined by extending the position of the top SNP (with the most significant *P*-value) on either side so that the –log_10_ (*P*-value) of all SNPs in this region was higher than the –log_10_ (*P*-value) of the top SNP minus 2 units, or expressed by (−log_10_ P)−2, where *P* is the *P*-value of the top SNP. Taking into account the linkage disequilibrium (LD) of the genome and the regulatory patterns of genes, we also defined those genes with significant SNPs within 10 Kb upstream and downstream as candidate genes. Additionally, haplotype blocks containing significantly associated SNPs were analyzed using Haploview software (21) and protein structure predictions were performed at https://robetta.bakerlab.org/.

### Statistical Analyses

The R language tool (v4.0.2) was used to perform descriptive statistics of the phenotypic data. One-way analysis of variance (ANOVA) was used to analyze the overall data, and the *t*-test was used among the groups. Statistical significance was set at *P* < 0.05, and results with *P* < 0.01 were considered extremely significant.

## Results

### The Data on Beak Length in XGG

The beak of XGG is an important structure with different phenotypes. We measured the beak length of 772 XGG individuals from a conservation farm. We then carried out quality control for the phenotypes and removed individuals with missing phenotypes. We eventually retained 749 high-accuracy phenotypic samples (613 females and 136 males). Beak length ranged from 61.36 mm to 93.40 mm, with an average beak length of 74.15 mm (Figure 1B). The beak length of the females was remarkably shorter than that of the males (*p* = 2E−16), indicating that gender had a significant effect on beak length (Figure 1C). In addition, in light of the fact that the beak length was measured by three investigators separately, we analyzed the measurement results by group. The results showed that the measurement results varied between groups, but the differences were not significant (Figure 1D).

### The Most Significant QTL Affected Beak Length

The heritability of beak length was estimated as 0.53 in the XGG population. This indicates that beak length had a moderate level of heritability with the potential for genetic selection for this trait. Considering the factors affecting phenotypic traits, we added gender and measurement group as fixed-effect factors in the GWAS analysis model. The Q-Q plot (Supplementary Figure S2) showed that the results of this model were reasonable and reliable, and the false-positive results caused by population stratification had little effect on the integrity of the results.

**Figure 2:**
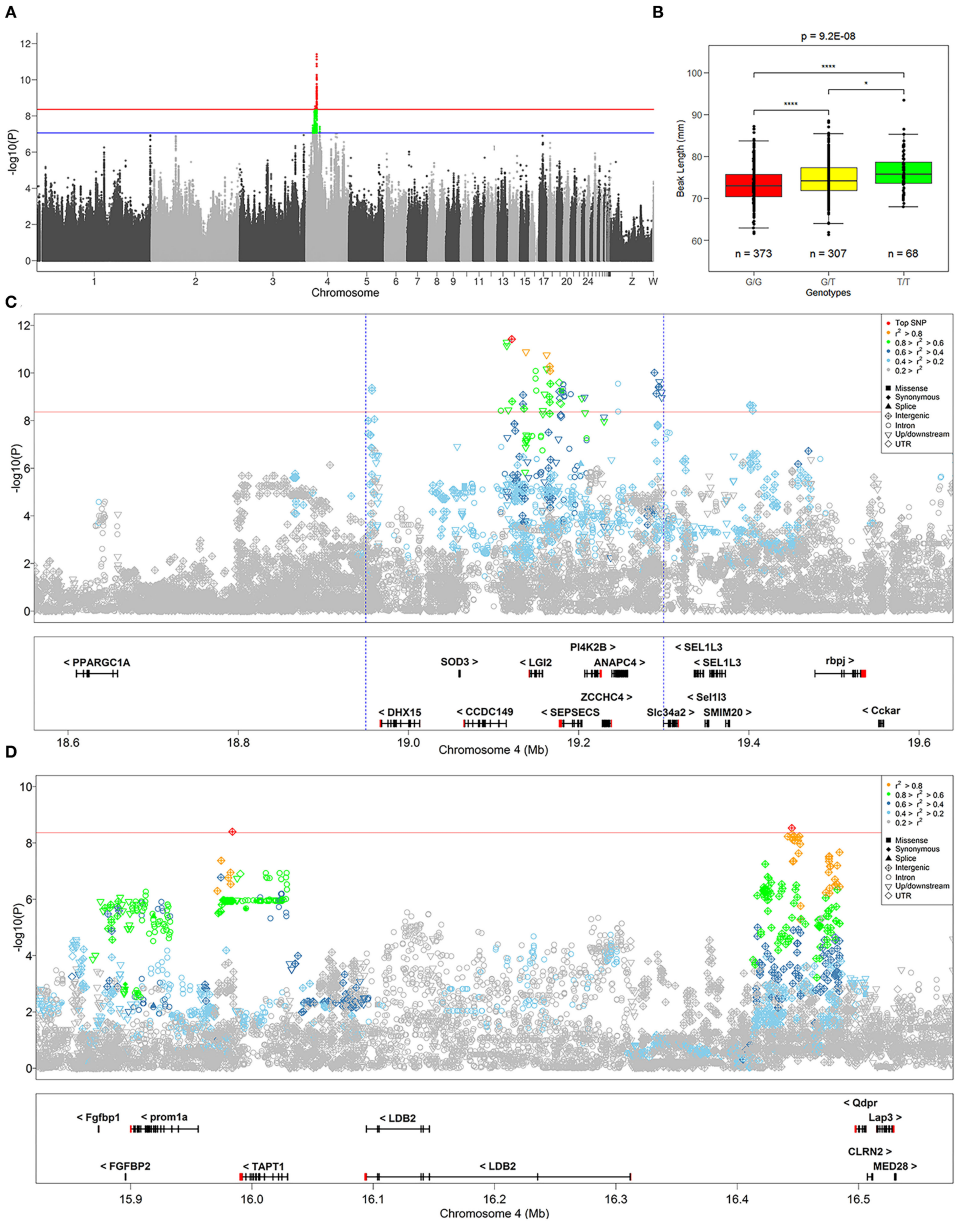
Summary of GWAS results. **(A)** Manhattan plot of GWAS for the beak length trait. The red and blue lines at points indicate significance thresholds at the genome and chromosome level, respectively. **(B)** Distribution of genotypes of the most significant SNP identified by GWAS in the beak length data. *Indicates *P* < 0.05, ****Indicates *P* < 0.01, and “*n*” indicates the number of samples. **(C,D)** Show the regional association plots of the loci of SNPs that are significantly associated with beak length. The red solid line indicates the significance threshold at the genome-wide level, and the defined most significant QTL region is marked with a blue dashed line. The difference in the color and shape of the points are shown as the result of *r*^2^ and variant annotation, respectively. The plot shows the names and locations of the genes, and the transcribed strand is indicated with an arrow. Genes are represented with intronic and exonic regions, and UTR regions are shown in red.

We identified 232 SNPs above the *P*-value threshold at the chromosome level (*P* = 8.69E−08) that were potentially affecting beak length trait (Supplementary Table S1). Among those, 57 achieved a *P*-value threshold at the genome-wide level (*P* = 4.34E−09) (Figure 2A). To identify the causal genes that affect the beak length of geese, we further analyzed the SNPs that reached the level of genome-wide association for confirmation and fine mapping of the globe locus, and identified the most significant QTL region within a 0.35 Mb interval (Chr 4: 18.95–19.30 Mb) that harbored 51 significant SNPs. Notably, the genotype of the top SNP (Chr 4:19121505, *P* = 3.89E-12) explained 7.24% of the phenotypic variation in beak length, which further illustrated that this region was likely to be a causal candidate region associated with the beak length trait in the XGG population.

### Candidate Genes and Mutations of Beak Length

To address the association of SNPs with the beak length trait, we performed region annotation and association analysis of candidate genes on all 232 SNPs that reached the chromosome-wide significance level (Supplementary Table S1). From this, twelve promising candidate genes were annotated, including *TBC1D1, BOD1L1, TAPT1, NCAPG, DHX15, CCDC149, LGI2, SEPSECS, ZCCHC4, ANAPC4, Slc34a2*, and *Rbm47*. Among them, seven genes (*TAPT1, DHX15, CCDC149, LGI2, SEPSECS, ANAPC4*, and *Slc34a2*) reached genome-wide significance levels (Figures 2C,D). The position of *TAPT1* (~16 Mb) was located far from the other six genes (~19 Mb).

To identify the strong candidate genes affecting the beak length of XGG, 57 SNPs at the genome-wide significance level were prioritized. Furthermore, we found that the visually remarkable region was divided into three QTLs, including a most significant QTL identified by the top SNP and two other low-effect QTLs (Figures 2C,D). In the most significant QTL region, we identified 51 SNPs at the genome-wide level involving five candidate genes (*DHX15, CCDC149, LGI2, SEPSECS*, and *ANAPC4*) associated with beak length (Figure 2C). Furthermore, we also assessed the degree of LD between the top SNP and surrounding SNPs. We found a low degree of LD (*r*^2^ <0.3) between the most significant SNPs identified in each QTL, which further confirms that they were different QTLs. However, the top SNP had a high degree of LD with its neighboring SNPs, with an average value of 0.62 (Table 1). We then tested for significant differences between pairwise genotypes of the top SNP (Figure 2B). Remarkable *P*-values were seen among three genotypes (*P* < 0.01), suggesting that it was likely that the top SNP was likely to be closely linked to the causal mutation affecting beak length. Interestingly, among these 51 SNPs, we found a missense mutation (Chr4: 4_19180040) in *SEPSECS* that resulted in a glutamine to lysine substitution, yet the presence of the mutation did not result in a substantial change in the overall structure of the protein (Supplementary Figure S3). However, the protein encoded by this gene converts O-phosphoseryl-tRNA (Sep) into selenocysteinyl-tRNA (Sec) to promote selenoprotein biosynthesis. This process might then affect the function of the brain by participating in neuronal development and further regulating beak development. Furthermore, we conducted haplotype block analysis for all significant SNPs. We found that these SNPs formed LD blocks, which were significantly associated with the beak length trait (Supplementary Figure S4). Therefore, it can be inferred from these analyses that beak length is jointly regulated by multiple genes, including *CCDC149, LGI2*, and *SEPSECS*.

**Table 1:** Summary of significant SNPs that are associated with the beak length trait.

**SNPs^a^**	***P*-value**	**Allele (major/minor)**	**MAF**	**LD (*r*^2^)^b^**	**Variant annotation^c^**	**Candidate genes^d^**
4_19121505	3.89E-12	G/T	0.30	1	Intergenic	*CCDC149*
4_19115564	5.30E-12	C/T	0.29	0.74	Upstream	*CCDC149*
4_19115596	7.45E-12	T/C	0.30	0.75	Upstream	*CCDC149*
4_19138017	1.33E-11	T/A	0.28	0.92	Downstream	*LGI2*
4_19162352	1.78E-11	G/A	0.30	0.86	Upstream	*LGI2*
4_19166083	5.43E-11	T/G	0.30	0.86	Intergenic	*LGI2*
4_19162397	7.03E-11	C/G	0.33	0.75	Upstream	*LGI2*
4_19166557	8.37E-11	T/G	0.30	0.87	Intergenic	*LGI2*
4_19149430	8.39E-11	T/C	0.23	0.71	Intron	*LGI2*
4_19289093	9.90E-11	A/G	0.35	0.51	Intergenic	*-*
4_19149283	1.74E-10	A/G	0.23	0.71	Intron	*LGI2*
4_19294723	2.37E-10	T/C	0.34	0.51	Upstream	*Slc34a2*
4_19177265	2.58E-10	C/T	0.25	0.66	UTR	*SEPSECS*
4_19246235	2.85E-10	G/A	0.29	0.35	Intron	*ANAPC4*
4_19166770	2.90E-10	A/G	0.25	0.66	Intergenic	*LGI2*
4_19182087	3.03E-10	T/A	0.22	0.56	Intron	*SEPSECS*
4_19181967	3.11E-10	C/T	0.23	0.56	Intron	*SEPSECS*
4_19294546	3.80E-10	G/C	0.34	0.50	Intergenic	*Slc34a2*
4_19294090	4.04E-10	G/A	0.34	0.51	Intergenic	*Slc34a2*
4_18957086	4.33E-10	T/A	0.15	0.37	Intergenic	*DHX15*
4_19181666	4.78E-10	T/C	0.22	0.56	Intron	*SEPSECS*
4_19150091	5.56E-10	C/T	0.22	0.67	Intron	*LGI2*
4_19150098	5.56E-10	A/G	0.22	0.67	Intron	*LGI2*
4_18957231	5.57E-10	A/G	0.14	0.34	Intergenic	*DHX15*
4_19180040	5.77E-10	T/G	0.34	0.65	Missense	*SEPSECS*
4_19177427	6.10E-10	T/C	0.23	0.57	UTR	*SEPSECS*
4_19297473	6.58E-10	G/A	0.34	0.5	Upstream	*Slc34a2*
4_19182183	6.73E-10	G/C	0.23	0.56	Intron	*SEPSECS*
4_19181913	6.87E-10	A/T	0.23	0.56	Intron	*SEPSECS*
4_19179134	6.89E-10	A/G	0.22	0.56	UTR	*SEPSECS*
4_19291403	7.54E-10	T/C	0.36	0.5	Intergenic	*Slc34a2*
4_19190667	7.85E-10	G/A	0.26	0.48	Intron	*SEPSECS*
4_19160006	7.91E-10	C/A	0.25	0.62	Upstream	*LGI2*
4_19134771	8.53E-10	A/G	0.16	0.48	Intergenic	*LGI2*
4_19183481	9.22E-10	G/A	0.23	0.6	Intron	*SEPSECS*
4_19183064	1.01E-09	A/G	0.22	0.56	Intron	*SEPSECS*
4_19165746	1.03E-09	A/G	0.33	0.8	Intergenic	*LGI2*
4_19206949	1.07E-09	G/A	0.24	0.52	Upstream	*SEPSECS*
4_19297635	1.10E-09	G/A	0.34	0.5	Upstream	*Slc34a2*
4_19178071	1.16E-09	G/A	0.23	0.56	UTR	*SEPSECS*
4_19203675	1.18E-09	C/G	0.24	0.63	Upstream	*SEPSECS*
4_19121824	1.58E-09	A/G	0.28	0.64	Intergenic	*CCDC149*
4_19166158	1.72E-09	G/A	0.32	0.79	Intergenic	*LGI2*
4_19178068	1.84E-09	C/A	0.37	0.71	UTR	*SEPSECS*
4_19179255	1.99E-09	G/C	0.34	0.65	UTR	*SEPSECS*
4_19134585	2.07E-09	A/G	0.16	0.48	Intergenic	*LGI2*
4_19401591	2.21E-09	C/T	0.44	0.35	Intergenic	-
4_19404666	2.40E-08	T/G	0.44	0.35	Intergenic	-
4_19401510	2.47E-09	T/G	0.45	0.35	Intergenic	-
4_16445056	2.95E-09	G/A	0.38	0.24	Intergenic	-
4_19135882	3.20E-09	A/T	0.34	0.71	Intergenic	*LGI2*
4_19117354	3.67E-09	A/G	0.23	0.71	Upstream	*CCDC149*
4_19158002	3.86E-09	G/A	0.23	0.72	Upstream	*LGI2*
4_19404633	3.88E-09	C/T	0.45	0.35	Intergenic	-
4_19108634	3.89E-09	G/A	0.27	0.62	Intron	*CCDC149*
4_15983935	3.99E-09	G/A	0.1	0.13	Intergenic	*TAPT1*
4_19246383	4.20E-09	T/C	0.3	0.32	Intron	*ANAPC4*

*MAF, minor allele frequency; LD, linkage disequilibrium*.

a *SNPs are expressed as chromosome_position*.

b *r^2^ represents the degree of LD between the top SNP and nearby SNPs*.

c *Variation annotation is supported by snpEff (v4.3t) software*.

d *The upstream and downstream influence range of a single gene was extended to 10 Kb to facilitate the detection of associated candidate genes when conducting association analyses.*

*“–”, Indicates that no candidate gene was found*.

## Discussion

The beak is an important feature of birds, but there are few reports on the genetic mechanism affecting its length. In this study, we studied the beak length of individuals of XGG, a representative local goose breed in China. By studying the variation in beak length in the XGG population, we found that the heritability of beak length was at an intermediate level; thus, it is possible to perform genetic selection for this trait. Based on GWAS analysis of the SNP datasets, seven candidate genes were identified at the genome-wide level, all of which are protein-coding genes. Homologs of the candidate gene *CCDC149* are reported to be expressed ubiquitously in the brain, suggesting that it plays an indispensable role in brain development (22). Although the exact function of this gene is still unclear and its effect on the beak has not been reported, it may have a previously unrecognized role in this process.

Both *LGI2* and *SEPSECS* are essential genes related to neurological disorders (23, 24), and they could affect development of the early nervous system. This in turn could affect the maturation of the brain and, thus, regulate the development of beak. All or most avian beaks consist of premaxillae bones that fuse during facial development in the embryo (25). The neural crest plays a vital role in head and face morphogenesis, as well as in bird beaks (26, 27). Establishment of the facial bones of birds, including the upper and lower jaw, is mediated by the neural crest, and the derived mesenchyme continues to interact with local tissues to form the characteristic patterns of the upper and lower beak (28). Neural crest mesenchyme also confers not only the shape and size of the beak, but also the density of the coracoid bone by regulating bone resorption (29). The beak of geese gradually forms in the direction of slenderness and broadness in the later stages of embryonic development (30). Waterfowl such as geese often need to immerse their heads in the water to catch food or overcome buoyancy when diving; thus, a slender and dense beak is an evolutionary adaptation to the aquatic environment.

The development of beak length is likely regulated by multiple genes and is mainly affected by the intracranial skeleton and osteoblasts. For example, there is evidence that *FGF13* may affect beak morphology by modulating the levels of osteoblasts (31). In comparison, the candidate gene *LGI2* identified in this study is comparable to *FGF13* as a key gene in synaptic transmission and may regulate the function of intracranial bone formation in the nervous system and brain, thereby affecting beak formation. In addition, *LGI2* also acts as a receptor to interact with some inactive ADAM proteins, thereby participating in processes in the brain (32). This could be another way for *LGI2* to participate in beak formation, because some ADAM proteins with catalytic activity play a role in cranial development by inducing neural crest cells in the Wnt signaling pathway (33).

Selenoprotein encoded by *SEPSECS* is involved in many physiological processes, especially in brain development. There is evidence that the synthesis of neuronal selenoproteins is essential for cerebellar development (34). Moreover, mutations in *SEPSECS* would lead to an overall selenoprotein deficiency, resulting in severe phenotypic variation (35). Previous studies have suggested that the beak and skull are highly integrated structures, and beak shape is linked to signaling molecules (36, 37). Therefore, we hypothesized that *SEPSECS* is involved in the regulation of brain function and plays a role in the regulation of beak length. Delay of telencephalic neurogenesis in the developmental stage is considered a prerequisite for vocalization learning in birds (38). Compared with songbirds, geese cannot expand their telencephalon by delaying telencephalic neurogenesis, which explains why the beaks of geese are better for preying than singing, and this may indicate that beak length is controlled jointly by the brain and neurogenesis.

The genes *TAPT1, DHX15, ANAPC4*, and *Slc34a2* are mainly related to the regulation of gene expression. *DHX15* is an ATP-dependent RNA helicase implicated in pre-mRNA splicing (39). *ANAPC4* is an E3 ubiquitin ligase that is regulated by the cell cycle, which mediates the ubiquitination and subsequent degradation of target proteins (40). *Slc34a2* is a phosphate transporter that may be involved in the transport of phosphate into cells via Na^+^ co-transport, providing sufficient phosphate for normal bone development (41). *TAPT1* is a transmembrane transporter that may be involved in the transduction or transmission of extracellular information needed for the axial skeletal pattern during development (42). In addition, *TAPT1* has also been reported to be involved in the development of craniofacial cartilage, which is related to the differentiation of cranial neural crest cells (43).

Overall, the candidate genes *CCDC149, LGI2*, and *SEPSECS* identified in this study are likely to be key genes that affect the beak length phenotype in geese. The brain is the most advanced part of the nervous system and its development determines the formation of the face, which undoubtedly has an important impact on the formation of beaks. Specifically, the key candidate genes identified in this study play a crucial role in the brain and nerves, which provides basic cognition for the formation and development of the beak.

## Conclusions

We conducted a GWAS for the beak length trait in the XGG population, based on SNPs obtained using whole-genome resequencing data. The analyses revealed that 57 SNPs on chromosome 4 were significantly associated with beak length at the genome-wide level, and seven candidate genes located near them were identified, namely *TAPT1, DHX15, CCDC149, LGI2, SEPSECS, ANAPC4*, and *Slc34a2*. Among them, *CCDC149, LGI2*, and *SEPSECS* might be the key genes affecting the beak length of XGG, especially the *LGI2* gene, which may play a vital role in the development of cranial neural crest cells and are highly relevant to the formation of geese beaks. The results of this study lay a foundation for studying the genetic mechanisms of phenotypic traits in geese, and also provide references for the molecular genetics of beak development in other avian species.

## Data Availability Statement

All raw resequencing data used in this study have been deposited into the NCBI BioProject database under accession PRJNA678815.

## Ethics Statement

The animal study was reviewed and approved by Jiangxi Science and Technology Normal University.

## Author Contributions

JH, XY, and HC conceived and designed the study. CW and HC analyzed the data. CW, JH, and HC wrote the paper. JO, HT, SZ, YX, YG, YW, and LW collected data and performed the sequencing. XY and JO provided funding support. All authors contributed to the article and approved the submitted version.

## Funding

This study was supported by the National Natural Science Foundation of China (No. 31860622 and 32060735).

## Conflict of Interest

The authors declare that the research was conducted in the absence of any commercial or financial relationships that could be construed as a potential conflict of interest.

## Publisher's Note

All claims expressed in this article are solely those of the authors and do not necessarily represent those of their affiliated organizations, or those of the publisher, the editors and the reviewers. Any product that may be evaluated in this article, or claim that may be made by its manufacturer, is not guaranteed or endorsed by the publisher.

## Supplementary Material

Supplementary Figure S1 Image of beak of XGG.

Supplementary Figure S2 Q-Q plot of beak length trait.

Supplementary Figure S3 The predicted 3-D structures of SEPSECS corresponding to a substitution of glutamine **(A)** to lysine **(B)** at position 420.

Supplementary Figure S4 Haplotype block analysis of all significant SNPs for beak length trait.

Supplementary Table S1 Summary of all detected significant SNP loci that associated with beak length trait. The red backgroud showed the SNPs above the genome-wide level.
