# Supplementary figures and images for: Identification of Key Candidate Genes for Beak Length Phenotype by Whole-Genome Resequencing in Geese
Source: Front Vet Sci. 2022 Mar 15;9:847481. doi: 10.3389/fvets.2022.847481 (PMC8964941; doi:10.3389/fvets.2022.847481)

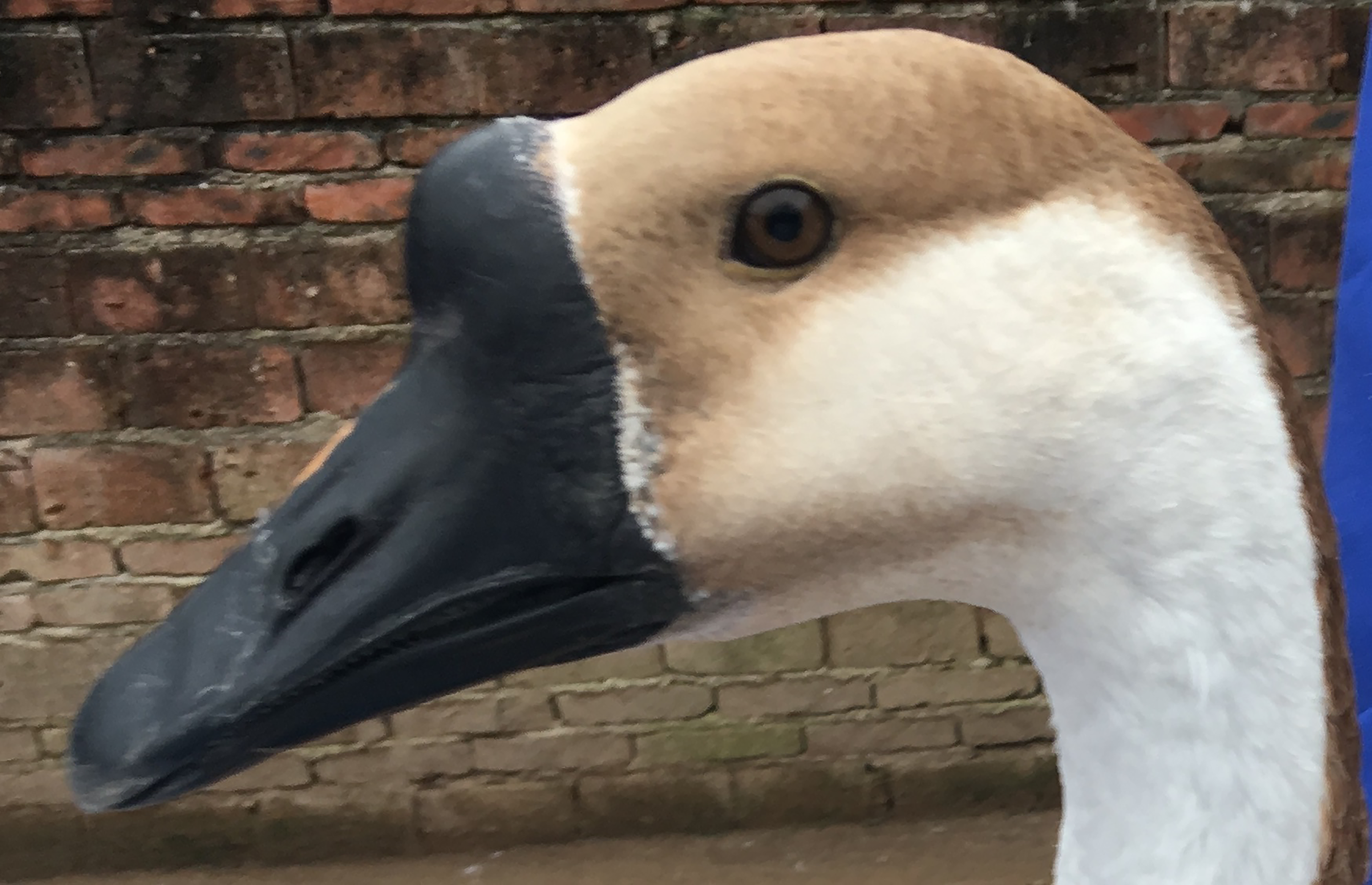

Supplement: Supplementary Figure S1 — Image of beak of XGG. [file Image_1.TIF]

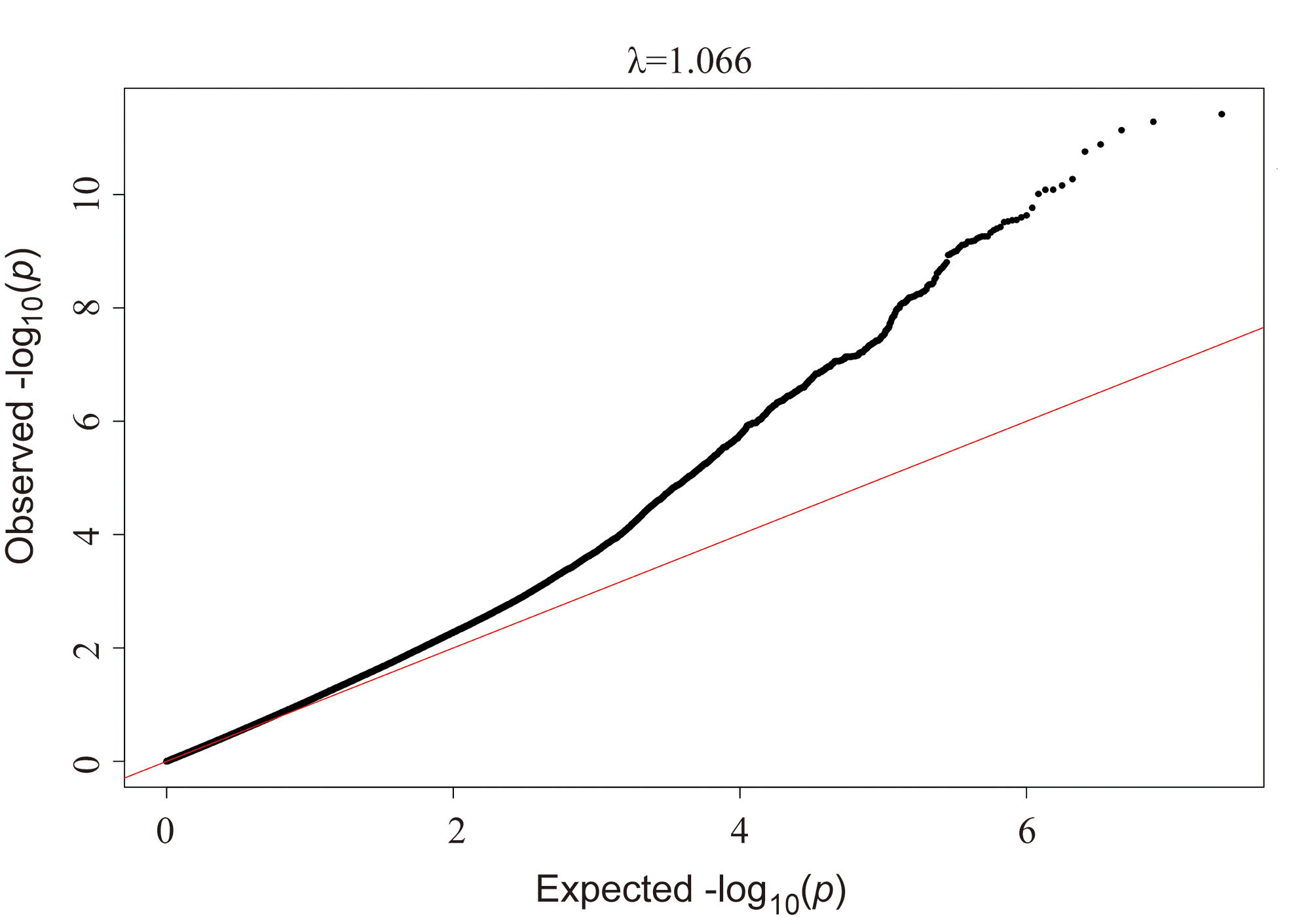

Supplement: Supplementary Figure S2 — Q-Q plot of beak length trait. [file Image_2.TIF]

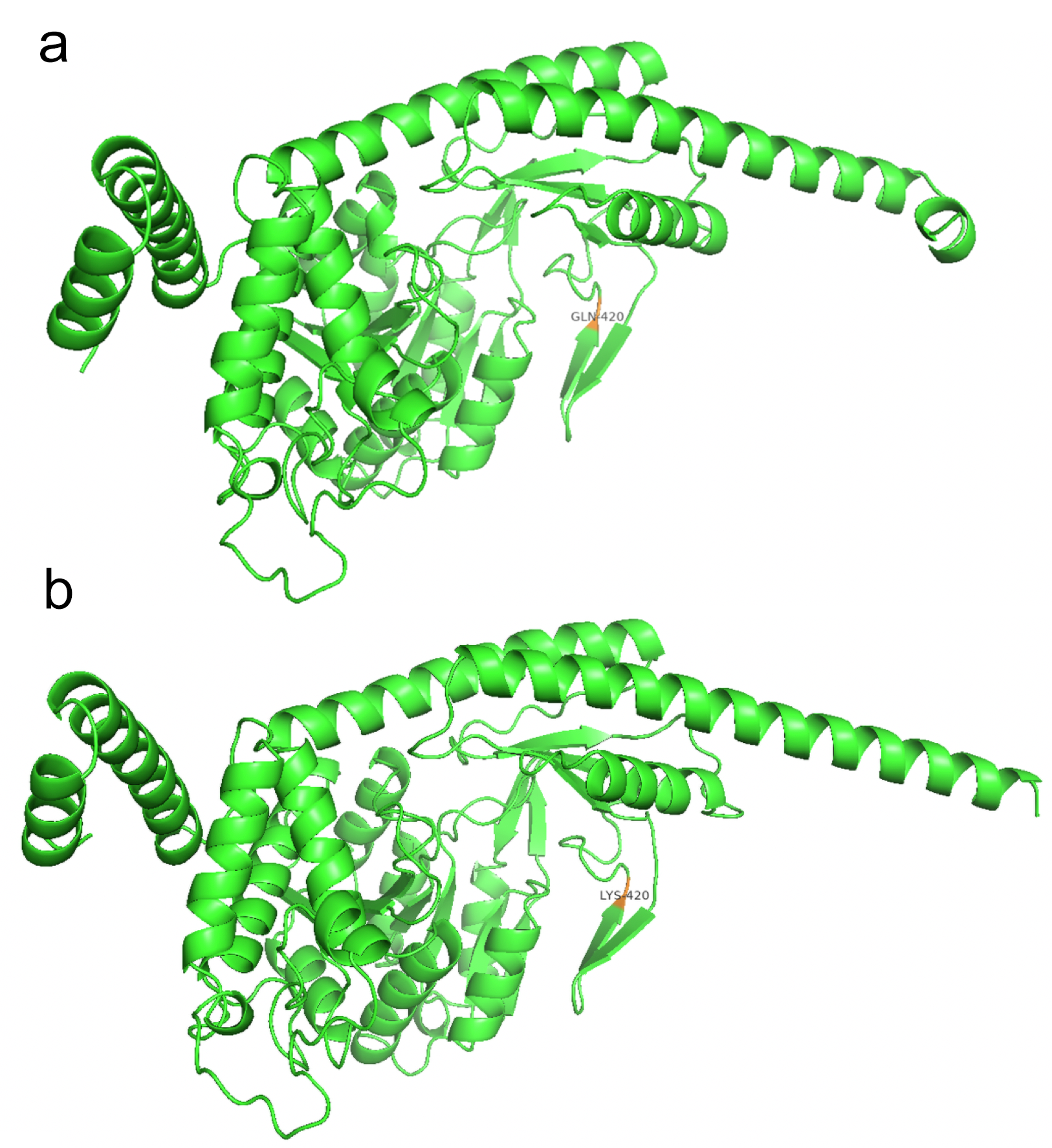

Supplement: Supplementary Figure S3 — The predicted 3-D structures of SEPSECS corresponding to a substitution of glutamine (A) to lysine (B) at position 420. [file Image_3.PNG]

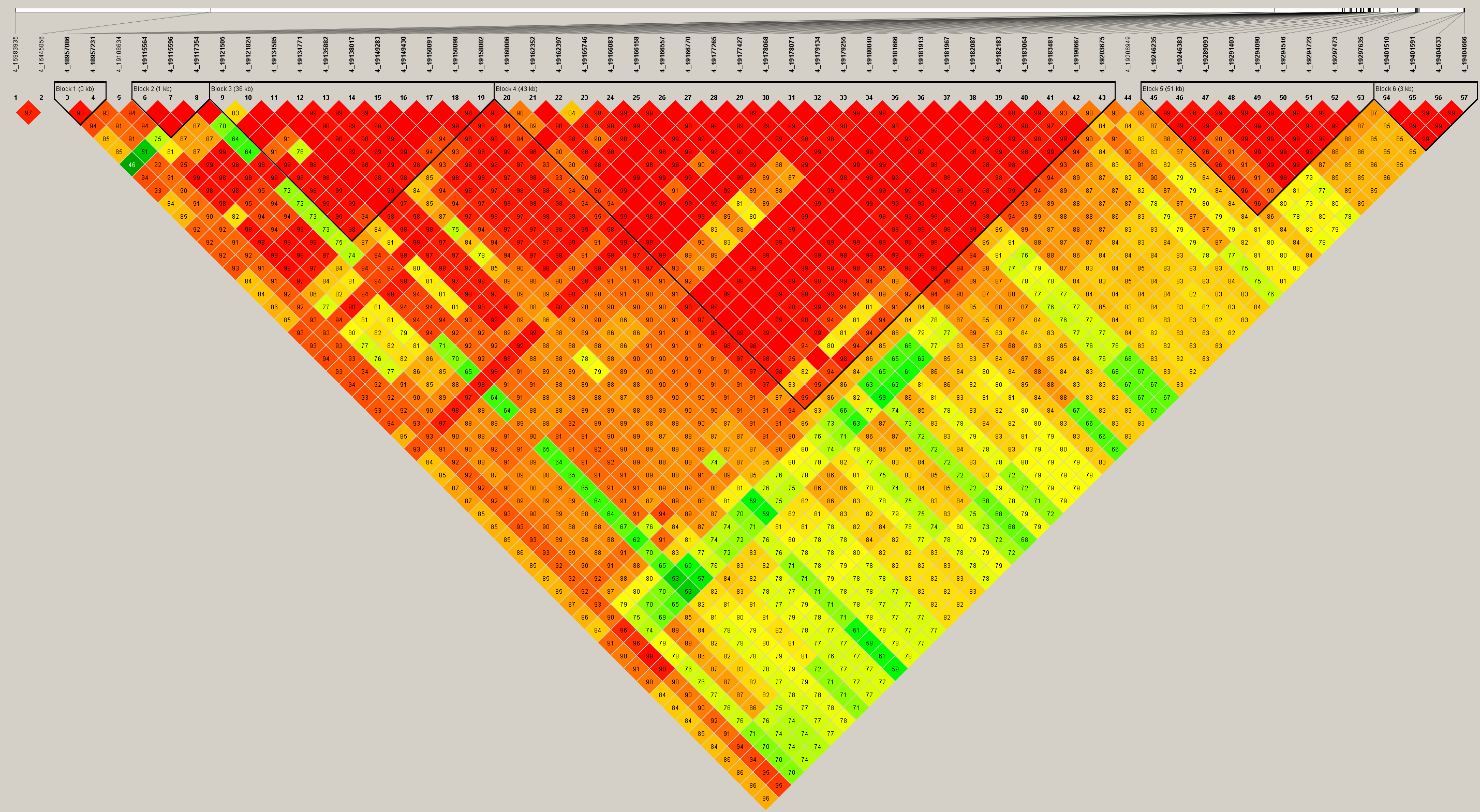

Supplement: Supplementary Figure S4 — Haplotype block analysis of all significant SNPs for beak length trait. [file Image_4.PNG]
